# Prevalence of Obesity and Associated Risk Factors and Cardiometabolic Comorbidities in Rural Northeast China

**DOI:** 10.1155/2019/6509083

**Published:** 2019-07-25

**Authors:** Shiwen Yu, Liying Xing, Zhi Du, Yuanmeng Tian, Li Jing, Han Yan, Min Lin, Boqiang Zhang, Shuang Liu, Yaping Pan, Chen Li

**Affiliations:** ^1^Department of Periodontics, School of Stomatology, China Medical University, Shenyang, Liaoning, China; ^2^Disease Control and Prevention of Liaoning Province, Shenyang, Liaoning, China; ^3^Department of Cardiovascular Medicine, The First Hospital of China Medical University, Shenyang, Liaoning, China; ^4^Department of Cardiovascular Medicine, Benxi Central Hospital, Benxi, Liaoning, China

## Abstract

**Objective:**

To investigate the epidemiological features of obesity in rural Northeast China.

**Design:**

This was a 2017–2018 cross-sectional study of 10,891 participants aged ≥40 years that was designed to investigate the prevalence of obesity in rural areas of Liaoning Province. Demographic data, biochemical parameters, and physical examinations were completed by well-trained personnel. Logistic regression analyses were then carried out to investigate independent risk factors and associated cardiometabolic comorbidities of obesity.

**Results:**

The proportions of general obesity only, central obesity only, and combined obesity were 1.0%, 31.3%, and 17.4%, respectively. Overall, 49.8% of our subjects were obese. Female gender, being married, being separated/divorced/widowed, or eating more meat were significantly associated with obesity. Smoking, higher family income, or regular physical exercise were negatively associated with obesity. General obesity only was significantly correlated with hypertension, diabetes, and high triglycerides (OR = 2.79, OR = 2.79, and OR = 3.37, resp.). General obesity only was irrelevant to high total cholesterol, low high-density lipoprotein cholesterol, and high low-density lipoprotein cholesterol, although central obesity only, or combined obesity, was relevant to these factors. Prehypertension and prediabetes showed a positive association with different types of obesity.

**Conclusions:**

We identified a high prevalence of general and central obesity in rural Northeast China, with similar independent risk factors. Participants with combined obesity had the highest risk of cardiometabolic comorbidities, indicating that the combined use of both waist circumference and body mass index is useful in practice.

## 1. Introduction

Obesity is now a worldwide epidemic, with an estimated 57.8% of adults worldwide expected to be classified as obese by 2030 according to figures released by the World Health Organization (WHO) [1]. Obesity is characterized by an excessive accumulation of body fat that gives rise to significant comorbidities, such as diabetes, hypertension, dyslipidemia, cardiovascular disease, and many cancers [2–4]. Therefore, obesity is invariably referred to as a crucial public health problem that requires urgent attention in order to prevent obesity-related health outcomes.

Although body mass index (BMI) is a simple index that is commonly used for classifying obesity, it has inherent limitations that can lead to some obese individuals being disregarded [5]. Recently, evidence has been growing to suggest that an additional indicator of central obesity, waist circumference (WC), may be more closely associated with metabolic risks compared to BMI because it reflects body fat distribution and upper body adiposity [6, 7]. However, this remains debatable as a recent study reported that the predictive values of BMI and WC for obesity were equivalent [8]. Consequently, there is a suggestion that both BMI and WC should be taken into consideration and adopted when investigating the associations between obesity and risk factors. We believe that the combined use of both WC and BMI allows more accurate monitoring of obesity.

The prevalence of obesity is known to vary significantly across the world [9]. It is therefore important to design region-specific public health policies; this requires the collection of epidemiological data relating to obesity from different geographical areas. Although previous observations have reported the prevalence of obesity in rural northeast China [10, 11], the criteria used to define obesity in these previous articles might not be appropriate for the Asian population. Moreover, these previous articles described the prevalence of general obesity (general obesity only + combined obesity) and central obesity (central obesity only + combined obesity) but did not focus upon the prevalence of combined obesity. Consequently, these previous studies were unable to define the overall obesity rate. Based on a large sample size, the present study provides a recent and representative depiction of obesity in the rural Chinese population, including prevalence and associated factors, and it clarifies the influence of different types of obesity upon different comorbidities.

## 2. Methods

### 2.1. Study Participants

This cross-sectional study aimed to analyze the prevalence of obesity in rural areas of Liaoning Province and was undertaken between September 2017 and May 2018. A multistage stratified cluster sampling procedure was used to ensure that the sample was representative. In stage one, we randomly selected four countries from Liaoning Province (Chaoyang, Lingyuan, Liaoyang, and Donggang). In stage two, we randomly selected 19 rural villages from each of four counties. In the final stage, we included all permanent residents (those living in their current domicile for ≥ 1 year) aged ≥ 40 years from the chosen villages. Afterwards, we excluded subjects if they were pregnant or if they had a mental disorder.

This study was granted ethics approval by the Central Ethics Committee at the China National Center for Cardiovascular Disease. We obtained written informed consent from all study participants.

### 2.2. Data Collection and Measurement

Data were collected by a team consisting of trained specialists in the prevention and control of chronic disease, dedicated cardiologists, and neurologists. The team used a standard questionnaire in a face-to-face interview to acquire data during a single clinic visit. All team members underwent rigorous knowledge training before the investigation and completed pilot studies on volunteers to specific standards prior to commencing the research study.

Demographic and clinical data, including data on age, sex, socioeconomic status (education, occupation, and annual household income), lifestyle (smoking, drinking, and physical activity), comorbidities (such as hypertension, diabetes, dyslipidemia, stroke, and heart disease), and other medical histories (such as coronary artery disease and cerebrovascular disease), were collected through face-to-face interview. To ensure that the results were recorded truthfully and carefully with an accuracy standard of at least 98%, the questionnaires were scanned, and relevant information was manually abstracted by trained staff, with double-entry as a quality check.

Physical data, including height, weight, and WC, were obtained during the interview. These parameters were measured to the nearest 0.1 kg in the case of weight and 0.1 cm in the case of height and WC, with participants wearing lightweight clothes without shoes. BMI was calculated by dividing weight in kilograms by height in meters squared (kg/m^2^). A central steering committee, with a subcommittee for quality control, was formed to ensure that all data were obtained in line with the standardized protocols.

Fasting blood samples were collected from all participants after at least 8 h of overnight fasting. Blood samples were obtained from an antecubital vein using BD Vacutainer tubes containing ethylenediaminetetraacetic acid (EDTA, Becton, Dickinson and Co., Franklin Lakes, NJ, USA); we then isolated serum from the whole blood for analysis. Then, all serum samples were frozen at -20°C for testing at a qualified laboratory. Biochemical parameters, such as fasting blood glucose (FBG), glycosylated hemoglobin (HbA-1c), serum lipid profiles including total cholesterol (TC), triglyceride (TG), serum high-density lipoprotein cholesterol (HDL-C), and serum low-density lipoprotein cholesterol (LDL-C), along with chemistry laboratory tests (liver function test, renal function test, electrolyte, calcium, and phosphorus level), were measured using an Abbott Diagnostics C800i autoanalyzer (Abbott Laboratories, Abbott Park, Illinois, USA) and commercial kits. We randomly selected 10% of specimens from each site for centralized testing at the Ministry of Health's Center for Clinical Laboratory to ensure that appropriate and reproducible laboratory standards were achieved.

For each participant, we measured blood pressure three times, at two-minute intervals, after at least five minutes of rest in a seated position using a standardized automatic electronic sphygmomanometer (J30, Omron, Kyoto, Japan). During the interview, participants were asked if they had used or taken any prescription drugs for blood pressure, lipid, or glucose control in the previous 2 weeks. Those who answered “yes” were asked to report the name, dose, and frequency of each drug, if they knew the drug names. Those who did not remember the exact dose were asked to clarify the number of tablets taken. We cleaned the data and then checked the generic names of the medication against China Pharmacopoeia 2015, with a 95% success rate.

### 2.3. Definitions

In terms of the criteria recommended by the Working Group on Obesity in China [12], general obesity is defined as a BMI ≥ 28 kg/m^2^. Based on the guidelines of the International Diabetes Federation for Chinese populations [13], central obesity is defined as a WC ≥ 90 cm for men and ≥ 80 cm for women. To carry out detailed international comparisons, additional BMI cut-off points and WC cut-off points were also applied. Prehypertension was defined as a mean systolic blood pressure (SBP) of 120–139 mmHg or mean diastolic blood pressure (DBP) of 80–89 mmHg when a subject was not taking antihypertensive medication [14]. Hypertension was defined as a mean SBP ≥ 140 mmHg or a mean DBP ≥ 90 mmHg and/or self-reported use of antihypertensive medication in the previous 2 weeks. Dyslipidemia was diagnosed if the participants met one of the following criteria: (1) serum TC level ≥ 6.22 mmol/L, (2) serum LDL-C level ≥ 4.14 mmol/L, (3) serum TG level ≥ 2.27 mmol/L, (4) serum HDL-C < 1.04 mmol/L, and (5) self-reported use of lipid-regulating medications over the previous 4 weeks. Elevated or decreased lipid status was determined according to the cut-off points mentioned above. Diabetes mellitus was diagnosed as an FBG ≥7.0 mmol/L or HbA1c ≥6.5%, and/or self-reported diagnosis that was previously determined by a physician. Prediabetes was defined when participants did not have diabetes but had an FBG level of 5.6–6.9 mmol/L and/or an HbA1c level of 5.7–6.4% [15].

### 2.4. Statistical Analysis

Descriptive statistics were calculated for all variables. Continuous variables are presented as means and standard deviations (SDs). Categorical variables are expressed as numbers or percentages. Means and proportions were calculated for population characteristics by gender. Differences in these characteristics were compared using Student's t test or the *χ*^2^ test. Prevalence rates of the various types of obesity stratified by gender and age were determined, and comparisons between men and women were carried out using the *χ*^2^ test. The adjusted odds ratio (OR) and 95% confidence interval (CI) of developing general or central obesity were determined from stepwise logistic regression models that included gender, age group, current smoking status, current drinking status, educational level, annual income, marital status, diet, and regular physical exercise. Multivariate logistic regression analyses were also carried out to investigate cardiometabolic comorbidities associated with general and central obesity. All statistical analyses were performed using SPSS software version 22.0 (SPSS Inc., Chicago, IL, USA) and* P *values < 0.05 were considered to be statistically significant.

## 3. Results

### 3.1. Baseline Characteristics of Study Participants

In our report, 12,808 individuals were selected and invited to participate in the survey, and 10,926 (85.3%) individuals completed the study. We further excluded participants (n = 35) for the following reasons: missing blood specimens (n = 31) and abnormal laboratory data (n = 4). Eventually, data from a total of 10891 (99.7%) subjects (4375 men and 6516 women) were analyzed.

The participants' characteristics by gender are listed and the distributions of characteristics of 4,375 men and 6,516 women are shown in Table 1. The participants' age ranged from 40 to 101 years with a mean age of 61.0 ± 10.1 years for men and 59.2 ± 10.0 years for women. Men had significantly higher DBP, cigarette consumption, alcohol consumption, educational levels, family income, unmarried rate, meat consumption and physical activity levels, and lower HbA1C, TC, TG, LDL, HDL, and BMI compared to women (all* P* < 0.05).

### 3.2. Prevalence of Obesity

**Figure 1:**
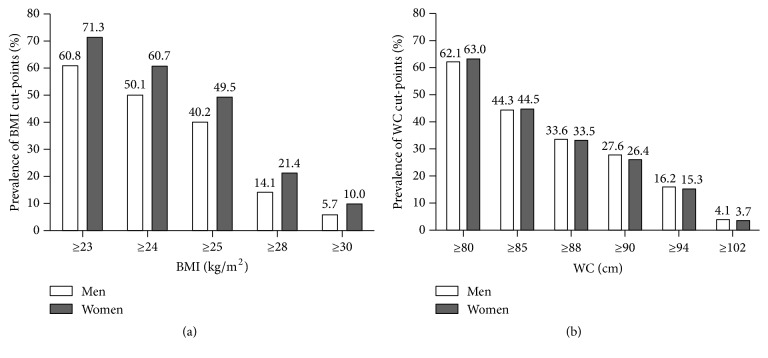
Prevalence of BMI (a) and WC (b) cut-off points in rural Northeast China. Abbreviations: BMI, body mass index; WC, waist circumference.

Table 1 shows that the prevalence of general obesity was 18.5% (men 14.1% and women 21.4%). The prevalence of central obesity was 48.7% (women 27.6% and men 63.0%). To carry out detailed international comparisons, data were analyzed by multiple BMI categories (≥ 23, ≥ 24, ≥ 25, ≥ 28, and ≥ 30) and WC cut-off points (≥ 80, ≥ 85, ≥ 88, ≥ 90, ≥ 94, and ≥ 102) (Figure 1).

In view of BMI and WC indicators, obese participants were reclassified into three types, including general obesity only (high BMI with normal WC), central obesity only (high WC with normal BMI), and combined obesity (high BMI with high WC). The prevalence of these various types of obesity by gender and age are shown in Table 2. The proportions of our study cohort with general obesity only, central obesity only, and combined obesity were 1.0%, 31.3%, and 17.4%, respectively. Overall, 49.8% of our subjects were obese. Besides that, the overall prevalence of general obesity only, central obesity only, and combined obesity were 1.9%, 15.3%, and 12.3% for men and 0.5%, 42.1%, and 20.9% for women, respectively (*P *< 0.001).

### 3.3. Factors Associated with General and Central Obesity

Stepwise logistic regression analysis was carried out to analyze correlations between obesity and demographic characteristics (Table 3). Female gender was significantly associated with both general obesity (OR = 1.27,* P *< 0.001) and central obesity (OR = 3.73,* P *< 0.001). General obesity was more common among participants aged 40–49 years compared with participants aged ≥ 60 years (60–69 years (OR = 0.70,* P *< 0.001), 70–79 years (OR = 0.47,* P *< 0.001), or ≥ 80 years (OR = 0.15,* P *< 0.001)). Participants aged 50–59 and 60–69 years were more prone to central obesity compared with those aged 40–49 years (OR = 1.28 and 1.25, resp.). Current smokers were less likely to be generally or centrally obese. Education at high school or above was associated with central obesity. Participants who were married (general obesity: OR = 2.89,* P *= 0.003; central obesity: OR = 2.52,* P *< 0.001) or separated/divorced/widowed (general obesity: OR = 2.93,* P *= 0.003; central obesity: OR = 2.77,* P *< 0.001) were more likely to be obese than those who had never been married. In addition, a higher income, eating more vegetables, and regular physical exercise were associated with lower risks of general and central obesity.

### 3.4. Various Types of Obesity and Cardiometabolic Comorbidities

Table 4 shows the relationships between different obesity groups and various cardiometabolic comorbidities. Compared with the nonobese group, the ORs for hypertension, diabetes, and high TG were increased in the general obesity only group (OR=2.79,* P* < 0.001, OR = 2.79,* P *< 0.001, and OR = 3.37,* P* <0.001, resp.), but the ORs for high TC, low HDL-C, and high LDL-C were not. Nevertheless, the central obesity only and combined obesity groups showed a progressive increase in the OR for each comorbidity type.In addition, we further analyzed the effects of different obesity groups on prehypertension and prediabetes, and we found that the three groups showed a progressive increase in OR (general obesity only: OR = 3.78 and 1.64; central obesity only: OR = 1.82 and 1.40; combined obesity groups: OR = 3.58 and 1.94, resp.).

## 4. Discussion

The current study investigated the prevalence of various types of obesity using population-specific cutoffs for the Chinese population in rural Northeast China. The results showed that the levels of obesity are still unacceptably high, despite the fact that effective measures may have been taken to control the upward trend [16, 17]. General and central obesity were associated with very similar risk factors, a fact that will assist in preventive obesity planning. Different types of obesity showed significant effects on the risk of cardiometabolic comorbidities, and combined obesity showed the highest risk. Consequently, taking measures to control obesity will help to reduce the risks of comorbidities.

Because Asians tend to have a relatively high risk of obesity-linked diseases using lower cut-off criteria [18], we reported the prevalence rate based on lower cut-off points for BMI and WC. We identified a prevalence rate of 18.5% for general obesity and 48.7% for central obesity in the population aged ≥ 40 years in rural Northeast China. In a previous study, Guo et al. [11] reported that the prevalence of general obesity (BMI ≥ 30 kg/m^2^) and central obesity (WC  ≥  102 cm for males and WC  ≥  88 cm for females) was 7.8% and 15.1%, respectively, in northeast China for the 2013–2014 period. Using exactly the same criteria, the prevalence in our study was 8.3% and 21.7%, respectively. Accordingly, the prevalence of obesity in rural Northeast China has experienced swift growth over a short 5-year period. The major reason for this may be that health resources in the rural areas of northeast China are relatively poor. Currently, township hospitals need to expand their work from medical services to preventive services, from individual services to group services, from technical services to social services, and from physical services to psychological services.

Similarly, the prevalence of obesity was different in our study area compared to the prevalence in other regions of China; this may be attributable to different cut-off points, age distributions, and the study population itself. A previous cross-sectional survey of 15,364 participants aged ≥ 15 years in Jiangxi Province demonstrated that the prevalence of general obesity (BMI ≥ 28 kg/m^2^) was 7.9% and the prevalence of central obesity (WC ≥ 95/90 cm) was 10.2% [19]. A possible explanation for this difference may be the different age distributions. Additionally, the prevalence of obesity in different populations is known to be different. For example, 11.2% and 59.4% of Chinese hypertensive adults aged 47–75 years in Jiangsu were diagnosed as having general obesity (BMI ≥ 30 kg/m^2^) and central obesity (WC ≥ 90/80 cm), respectively [20].

Considering the BMI and WC categories, obesity can be classified into three types: general obesity only, central obesity only, and combined obesity. Our study showed that the prevalence of general obesity only, central obesity only, and combined obesity were 1.0%, 31.3%, and 17.4%, respectively. The overall obesity rate was 49.8%. By calculation, approximately 62.4% of obese subjects would be neglected if WC was not used to identify obesity, which is consistent with a previous study [21]. This is an indication that WC can outperform BMI in providing obesity-related information, and measures of WC should be used, in addition to BMI, in the clinical evaluation of obesity.

In our present study, we used logistic regression analyses to investigate the associations between obesity and demographic characteristics. From 40 to 69 years old, the risk of central obesity grew gradually. Since body fat is inclined to accumulate in the abdomen after middle age [22], this results in a large proportion of individuals developing central obesity. Interestingly, although there is good evidence that the prevalence of general obesity increases with age [19], an inverse relationship was observed in people > 60 years old. This might be due partly to obesity-related mortality [23]. In addition, our current study reported that female gender was significantly associated with general and central obesity in rural Northeast Chinese adults; this was also reported previously [11, 24]. Gender differences in terms of developing obesity may be due to the fact that men generally engage in exceedingly hard physical labor in rural areas. Furthermore, the rapid changes in steroid hormone levels in women may be responsible for weight gain [25].

Even in this relatively poor population of rural Northeast China, lifestyle has also been found to be related to both general and central obesity. In the present study, transition into or out of marriage, lower family income, eating more meat, and lower physical activity levels were also independent risk factors for general and central obesity, as seen in previous studies [21, 26, 27]. Numerous lines of evidence indicate that lower educational attainment shows the strongest consistent relationship with obesity [28–30]. In contrast, the present study also showed that subjects with high school education or above had a higher risk of central obesity. The exact reasons for this are not clear. In rural China, well-educated subjects mainly engage in nonphysical work and invariably spend more time sitting in front of computers.

Most importantly, those who smoke cigarettes tend to have general and central obesity [31]. Interestingly, whether alcohol intake increases obesity remains controversial [32]. Recent studies showed that light-to-moderate alcohol consumption is not associated with adiposity gain, while heavy drinking is more consistently related to weight gain [33]. Our study showed that there were no associations between drinking and either general or central obesity. This may be because women were oversampled; furthermore, the amount and frequency of alcohol consumption were not defined specifically in our study.

In the current study, as with previous studies [11, 34, 35], the effects on the development of hypertension and diabetes of general obesity seemed to be enhanced by combined obesity. Even more interesting is the fact that general obesity only was not associated with an increased risk of high TC, low HDL-C, and high LDL-C, while central obesity only and combined obesity were. This implies that combining general and central obesity to evaluate the development of cardiometabolic comorbidities was more beneficial than considering single effects alone. This is mainly explained by the fact that the atherogenic lipoprotein profile of a viscerally obese individual includes an increased proportion of small LDL and a reduced concentration of large HDL particles [22]. More research effort is now needed to identify the mechanism(s) underlying these observed phenomena. The perceived potential for cardiovascular disease risk enhancement by prehypertension and prediabetes in healthy Chinese adults has already been confirmed [36]; we also observed that prehypertension and prediabetes had a positive association with different types of obesity. Thus, BMI and WC should be detected earlier in adults in order to reduce the prevalence of cardiometabolic comorbidities.

*Study Limitations*. Our study has some limitations which need to be considered. First, the cross-sectional design of our study only allowed the assessment of the associations between obesity and risk factors or comorbidities rather than causal links. Second, although the study included a large number of subjects, these participants were all from the northeast of China and those > 40 years old. This may reduce the applicability of our results to other populations. Third, substantial absolute errors in measuring blood pressure, height, weight, and WC may have occurred [37]. Other limitations include potential misclassification related to recall bias and confounding factors.

## 5. Conclusions

Our present data highlight the high prevalence of general and central obesity in rural northeastern China and the two types of obesity had similar independent risk factors. Our results also suggest that public health programs are required to promote balanced diets and regular physical exercise and thus overcome the obesity epidemic. Furthermore, participants with combined obesity had the highest risks of cardiometabolic comorbidities compared to single obesity, implying that both BMI and WC could be used in practice to evaluate the presence of elevated health risk.

## Figures and Tables

**Table 1:** Population characteristics by sex, n (%).

	Men	Women	*P* value^d^
N	4375	6516	
Age (y)^a^	61.0, 10.1	59.2, 10.0	<0.001
Age group (y)			
40-49	655(15.0)	1220(17.2)	<0.001
50-59	1216(27.8)	2058(31.6)	
60-69	1619(37.0)	2207(33.9)	
70-79	742(17.0)	863(13.2)	
≥80	143(3.3)	168(2.6)	
SBP (mm Hg)^a^	145.4, 22.1	145.9, 24.6	0.272
DBP (mm Hg)^a^	87.9, 11.7	85.9, 12.0	<0.001
FBG (mmol/L)^a^	6.2, 1.8	6.2, 1.9	0.788
HbA1c (%)^a^	5.5, 0.9	5.7, 1.2	<0.001
TC (mmol/L)^a^	4.9, 1.0	5.2, 1.1	<0.001
TG (mmol/L)^a^	1.6, 1.5	1.7, 1.5	<0.001
LDL (mmol/L)^a^	2.3, 0.9	2.4, 1.1	<0.001
HDL (mmol/L)^a^	1.9, 0.8	2.0, 0.8	<0.001
BMI (kg/m^2^)			
Mean^a^	24.3, 3.6	25.2, 3.9	<0.001
General obesity^b^	619(14.1)	1394(21.4)	<0.001
WC (cm)			
Mean^a^	83.5, 10.3	83.3, 10.2	0.216
Central obesity^c^	1206(27.6)	4103(63.0)	<0.001
Current smoking	2409(55.1)	469(7.2)	<0.001
Current drinking	2430(55.5)	633(9.7)	<0.001
Hypertension	2680(61.3)	3919(60.1)	0.244
Diabetes	668(15.3)	1098(16.9)	0.028
Heart disease	220(5.0)	363(5.6)	0.218
Stroke	375(8.6)	361(5.5)	<0.001
Dyslipidemia	1213(27.7)	2163(33.2)	<0.001
Educational level			
Primary school or below	2140(48.9)	4302(66.0)	<0.001
Middle school	1676(38.3)	1806(27.7)	
High school or above	559(12.8)	408(6.3)	
Annual income (CNY/year)			
≤5000	1731(39.6)	3982(45.8)	<0.001
5001-10000	980(22.4)	1384(21.2)	
10000-20000	790(18.1)	1176(18.0)	
>20000	874(20.0)	974(14.9)	
Marital status			
Unmarried	135(3.1)	5(0.1)	<0.001
Married	3847(87.9)	5712(87.7)	
Divorced, widowed, other	393(9.0)	799(12.3)	
Diet			
Balanced	1984(45.3)	2724(41.8)	<0.001
More meat	876(20.0)	533(8.2)	
More vegetables	1515(34.6)	3259(50.0)	
Regular physical exercise			
Yes	3716(84.9)	5371(82.4)	<0.001
No	659(15.1)	1145(17.6)	

Abbreviations: SBP, systolic blood pressure; DBP, diastolic blood pressure; TC, total cholesterol; TG, triglyceride; LDL-C, low-density lipoprotein cholesterol; HDL-C, high-density lipoprotein cholesterol; FPG, fasting plasma glucose; HbA1c, hemoglobin A1c; BMI, body mass index; WC, waist circumference; CNY, China Yuan (1CNY = 0.161 USD).

^a^Data represent Mean Standard Deviation.

^b^General obesity were defined as a BMI of ≥ 28.0 kg/m^2^.

^c^Central obesity was defined as a waist circumference ≥ 90 cm for men and ≥ 80 cm for women.

^d^ *P* values are calculated for gender difference by t-test or *χ*2 test.

**Table 2:** Prevalence of various types of obesity stratified by gender and age.

Gender	Age group (y)	n	General obesity only	Central obesity only	Combined obesity	Total
Men	40–49	655	25(3.8)	90(13.7)	132(20.2)	247(37.7)
	50–59	1216	23(1.9)	190(15.6)	177(14.6)	390(32.1)
	60–69	1619	22(1.4)	254(15.7)	179(11.1)	455(28.1)
	70–79	742	11(1.5)	108(14.6)	44(5.9)	163(22.0)
	≥80	143	0(0.0)	26(18.2)	6(4.2)	32(22.4)
	Total	4375	81(1.9)	668(15.3)	538(12.3)	1287(29.4)
Women	40–49	1220	9(0.7)^*∗∗*^	403(33.0)^*∗∗*^	265(21.7)	677(55.5)^*∗∗*^
	50–59	2058	13(0.6)^*∗*^	849(41.3)^*∗∗*^	474(23.0)^*∗∗*^	1336(64.9)^*∗∗*^
	60–69	2207	10(0.5)^*∗*^	1021(46.3)^*∗∗*^	455(20.6)^*∗∗*^	1486(67.3)^*∗∗*^
	70–79	863	1(0.1)^*∗*^	377(43.7)^*∗∗*^	158(18.3)^*∗∗*^	536(62.1)^*∗∗*^
	≥80	168	0(0.0)	92(54.8)^*∗∗*^	9(5.4)	101(60.1)^*∗∗*^
	Total	6516	33(0.5)^*∗∗*^	2742(42.1)^*∗∗*^	1361(20.9)^*∗∗*^	4136(63.5)^*∗∗*^

Data represent n (%).

Gender difference ^*∗*^*P*< 0.05, ^*∗∗*^*P* < 0.001.

**Table 3:** Adjusted odds ratios^a^ (95% confidence intervals) of having general obesity^b^ and central obesity^c^.

Variables	General obesity		Central obesity	
	OR (95% CI)	*P* value	OR (95% CI)	*P* value
Sex, women	1.27(1.12-1.44)	<0.001	3.73(3.37-4.14)	<0.001
Age (y)				
40–49	1.00(ref)		1.00(ref)	
50–59	0.90(0.78-1.04)	0.149	1.28(1.13-1.45)	<0.001
60–69	0.70(0.60-0.81)	<0.001	1.25(1.10-1.42)	0.001
70–79	0.47(0.39-0.58)	<0.001	0.89(0.75-1.05)	0.151
≥80	0.15(0.08-0.25)	<0.001	0.84(0.63-1.11)	0.219
Current smoking, yes	0.52(0.45-0.60)	<0.001	0.54(0.48-0.60)	<0.001
Current drinking, yes	Not included	-	Not included	-
Educational level				
Primary school or below	1.00(ref)		1.00(ref)	
Middle school	Not included	-	1.03(0.93-1.13)	0.581
High school or above	Not included	-	1.37(1.17-1.60)	<0.001
Annual income (CNY/year)				
≤5,000	1.00(ref)		1.00(ref)	
5,001–10,000	0.78(0.68-0.89)	<0.001	0.75(0.67-0.84)	<0.001
10,001–20,000	0.75(0.65-0.87)	<0.001	0.77(0.68-0.87)	<0.001
>20,000	0.90(0.78-1.05)	0.184	0.85(0.75-0.97)	0.018
Marital status				
Unmarried	1.00(ref)		1.00(ref)	
Married	2.89(1.45-5.75)	0.003	2.52(1.55-4.09)	<0.001
Separated/divorced/widowed	2.93(1.44-5.94)	0.003	2.77(1.68-4.56)	<0.001
Diet				
Balanced	1.00(ref)		1.00(ref)	
More meat	1.69(1.46-1.96)	<0.001	1.64(1.44-1.88)	<0.001
More vegetables	0.83(0.74-0.92)	0.001	0.84(0.76-0.91)	<0.001
Regular physical exercise, yes	0.78(0.68-0.90)	<0.001	0.89(0.79-0.996)	0.043

Abbreviations: OR, odds ratio; 95% CI, 95% confidence interval; CNY, China Yuan (1CNY = 0.161 USD); ref, reference group.

^a^All variables were included in the same models.

^b^General obesity was defined as a BMI ≥ 28.0 kg/m^2^.

^c^Central obesity was defined as a waist circumference ≥ 90 cm for men and ≥ 80 cm for women.

**Table 4:** Effects of various types of obesity on different cardiometabolic comorbidities.

	General obesity only		Central obesity only		Combined obesity	
	OR(95% CI)	*P* value	OR(95% CI)	*P* value	OR(95% CI)	*P* value
Hypertension	2.47(1.63-3.76)	<0.001	1.78(1.61-1.96)	<0.001	3.51(3.09-3.99)	<0.001
Diabetes	2.20(1.38-3.53)	0.001	2.03(1.78-2.31)	<0.001	2.79(2.42-3.22)	<0.001
High TC	1.55(0.88-2.72)	0.126	1.56(1.36-1.78)	<0.001	1.68(1.44-1.96)	<0.001
Low HDL-C	0.97(0.39-2.42)	0.943	2.18(1.76-2.71)	<0.001	3.24(2.59-4.04)	<0.001
High LDL-C	0.42(0.06-3.03)	0.387	3.10(2.45-3.93)	<0.001	3.38(2.61-4.38)	<0.001
High TG	2.19(1.38-3.48)	0.001	2.42(2.13-2.76)	<0.001	3.37(2.93-3.87)	<0.001
Prehypertension	3.78(1.31-10.88)	0.014	1.82(1.53-2.17)	<0.001	3.58(2.68-4.78)	<0.001
Prediabetes	1.64(1.05-2.56)	0.029	1.40(1.26-1.55)	<0.001	1.94(1.70-2.22)	<0.001

Abbreviations: OR, odds ratio; 95% CI, 95% confidence interval; TC, total cholesterol; TG, triglyceride; LDL-C, low-density lipoprotein cholesterol; HDL-C, high-density lipoprotein cholesterol.

*∗*Adjusted for age, sex, smoking status, drinking status, education, family income, marital status, diet, physical activity, blood pressure, fasting plasma glucose, HbA1C, and lipid status, with nonobese participants as the reference.

## Data Availability

The data used to support the findings of this study are available from the corresponding author upon request.

## References

[1] Esmaili H., Bahreynian M., Qorbani M. (2015). Prevalence of general and abdominal obesity in a nationally representative sample of Iranian children and adolescents: the CASPIAN-IV study. *Iranian Journal of Pediatrics*.

[2] Ortega F. B., Lavie C. J. (2018). Introduction and update on obesity and cardiovascular diseases 2018. *Progress in Cardiovascular Diseases*.

[3] Garg S. K., Maurer H., Reed K., Selagamsetty R. (2014). Diabetes and cancer: two diseases with obesity as a common risk factor. *Diabetes, Obesity and Metabolism*.

[4] Del Prato S., Raz I. (2013). Introduction to the 4th world congress on controversies to consensus in diabetes, obesity and hypertension (CODHy). *Diabetes Care*.

[5] Sgambat K., Roem J., Mitsnefes M. (2018). Waist-to-height ratio, body mass index, and cardiovascular risk profile in children with chronic kidney disease. *Pediatric Nephrology*.

[6] Sahakyan K. R., Somers V. K., Rodriguez-Escudero J. P. (2015). Normal-weight central obesity: implications for total and cardiovascular mortality. *Annals of Internal Medicine*.

[7] Zhang C., Rexrode K. M., Van Dam R. M., Li T. Y., Hu F. B. (2008). Abdominal obesity and the risk of all-cause, cardiovascular, and cancer mortality: 16 years of follow-up in US women. *Circulation*.

[8] Canoy D., Boekholdt S. M., Wareham N. (2007). Body fat distribution and risk of coronary heart disease in men and women in the european prospective investigation into cancer and nutrition in norfolk cohort. *Circulation*.

[9] Balkau B., Deanfield J. E., Després J. (2007). International day for the evaluation of abdominal obesity (IDEA): a study of waist circumference, cardiovascular disease, and diabetes mellitus in 168 000 primary care patients in 63 countries. *Circulation*.

[10] Zhang X., Sun Z., Zhang X. (2008). Prevalence and associated factors of overweight and obesity in a chinese rural population. *Obesity*.

[11] Guo X., Li Z., Guo L. (2014). An update on overweight and obesity in rural Northeast China: from lifestyle risk factors to cardiometabolic comorbidities. *BMC Public Health*.

[12] Zhou B.-F., Cooperative Meta-Analysis Group of the Working Group on Obesity in C (2002). Predictive values of body mass index and waist circumference for risk factors of certain related diseases in Chinese adults—study on optimal cut-off points of body mass index and waist circumference in Chinese adults. *Biomedical and Environmental Sciences*.

[13] International Diabetes Federation The IDF consensus worldwide definition of the metabolic syndrome. http://www.idf.org/webdata/docs/IDF_Meta_def_final.pdf.

[14] Chobanian A. V., Bakris G. L., Black H. R. (2003). The seventh report of the joint national committee on prevention, detection, evaluation, and treatment of high blood pressure: the JNC 7 report. *The Journal of the American Medical Association*.

[15] Marseglia A., Fratiglioni L., Kalpouzos G., Wang R., Bäckman L., Xu W. (2019). Prediabetes and diabetes accelerate cognitive decline and predict microvascular lesions: A population-based cohort study. *Alzheimer’s & Dementia*.

[16] Hawkes C., Smith T. G., Jewell J. (2015). Smart food policies for obesity prevention. *The Lancet*.

[17] Kegler M. C., Haardörfer R., Alcantara I. C. (2016). Impact of improving home environments on energy intake and physical activity: a randomized controlled trial. *American Journal of Public Health*.

[18] Aekplakorn W., Chongsuvivatwong V., Tatsanavivat P., Suriyawongpaisal P. (2011). Prevalence of metabolic syndrome defined by the international diabetes federation and national cholesterol education program criteria among thai adults. *Asia-Pacific Journal of Public Health*.

[19] Hu L., Huang X., You C. (2017). Prevalence of overweight, obesity, abdominal obesity and obesity-related risk factors in southern China. *PLoS ONE*.

[20] Qin X., Zhang Y., Cai Y. (2013). Prevalence of obesity, abdominal obesity and associated factors in hypertensive adults aged 45–75 years. *Clinical Nutrition*.

[21] Du T., Sun X., Yin P., Huo R., Ni C., Yu X. (2013). Increasing trends in central obesity among Chinese adults with normal body mass index, 1993–2009. *BMC Public Health*.

[22] Tchernof A., Després J.-P. (2013). Pathophysiology of human visceral obesity: an update. *Physiological Reviews*.

[23] Chang S., Yu Y., Carlsson N. P., Liu X., Colditz G. A. (2017). Racial disparity in life expectancies and life years lost associated with multiple obesity-related chronic conditions. *Obesity*.

[24] Wang H., Wang J., Liu M.-M. (2012). Epidemiology of general obesity, abdominal obesity and related risk factors in urban adults from 33 communities of northeast china: the CHPSNE study. *BMC Public Health*.

[25] Leeners B., Geary N., Tobler P. N., Asarian L. (2017). Ovarian hormones and obesity. *Human Reproduction Update*.

[26] Wang R., Zhang P., Gao C. (2016). Prevalence of overweight and obesity and some associated factors among adult residents of northeast China: A cross-sectional study. *BMJ Open*.

[27] Zapata M. E., Bibiloni M. D. M., Tur J. A. (2016). Prevalence of overweight, obesity, abdominal-obesity and short stature of adult population of Rosario, Argentina. *Nutrición Hospitalaria*.

[28] Ogden C. L., Fryar C. D., Hales C. M. (2018). Differences in obesity prevalence by demographics and urbanization in US children and adolescents, 2013-2016. *Journal of the American Medical Association*.

[29] Alves R. F. S., Faerstein E. (2015). Educational inequality in the occurrence of abdominal obesity: Pró-Saúde study. *Revista de Saúde Pública*.

[30] López-Sobaler A. M., Rodríguez-Rodríguez E., Aranceta-Bartrina J. (2016). General and abdominal obesity is related to physical activity, smoking and sleeping behaviours and mediated by the educational level: findings from the ANIBES study in Spain. *PLoS ONE*.

[31] Hong S., Cai Q., Chen D., Zhu W., Huang W., Li Z. (2012). Abdominal obesity and the risk of colorectal adenoma. *European Journal of Cancer Prevention*.

[32] Thomson C. A., Wertheim B. C., Hingle M. (2012). Alcohol consumption and body weight change in postmenopausal women: results from the Women's Health Initiative. *International Journal of Obesity*.

[33] Traversy G., Chaput J. (2015). Alcohol consumption and obesity: an update. *Current Obesity Reports*.

[34] Goh V. H., Hart W. G. (2015). Association of general and abdominal obesity with age, endocrine and metabolic factors in Asian men. *The Aging Male*.

[35] Zhang P., Wang R., Gao C. (2016). Prevalence of central obesity among adults with normal bmi and its association with metabolic diseases in Northeast China. *PLoS ONE*.

[36] Wu J., Yan W., Qiu L. (2011). High prevalence of coexisting prehypertension and prediabetes among healthy adults in northern and northeastern China. *BMC Public Health*.

[37] Kahn H. S. (2006). Obesity and risk of myocardial infarction: the INTERHEART study. *The Lancet*.

